# Equivariant spherical CNNs for accurate fiber orientation distribution estimation in neonatal diffusion MRI with reduced acquisition time

**DOI:** 10.3389/fnins.2025.1604545

**Published:** 2025-07-30

**Authors:** Haykel Snoussi, Davood Karimi

**Affiliations:** Department of Radiology, Boston Children's Hospital and Harvard Medical School, Boston, MA, United States

**Keywords:** diffusion MRI, spherical CNNs, neonatal brain, fiber orientation, geometric deep learning, tractography

## Abstract

Early and accurate assessment of brain microstructure using diffusion Magnetic Resonance Imaging (dMRI) is crucial for identifying neurodevelopmental disorders in neonates, but remains challenging due to low signal-to-noise ratio (SNR), motion artifacts, and ongoing myelination. In this study, we propose a rotationally equivariant Spherical Convolutional Neural Network (sCNN) framework tailored for neonatal dMRI. We predict the Fiber Orientation Distribution (FOD) from multi-shell dMRI signals acquired with a reduced set of gradient directions (30% of the full protocol), enabling faster and more cost-effective acquisitions. We train and evaluate the performance of our sCNN using real data from 43 neonatal dMRI datasets provided by the Developing Human Connectome Project (dHCP). Our results demonstrate that the sCNN significantly outperforms a Multi-Layer Perceptron (MLP) baseline across multiple quantitative metrics, including Mean Squared Error (MSE), Peak Signal-to-Noise Ratio (PSNR), Angular Correlation Coefficient (ACC), angular error, and peak match rate, indicating superior FOD estimation accuracy. More importantly, it yields FODs and tractography that are quantitatively comparable and qualitatively highly similar to those from a reliable Hybrid-CSD ground truth, despite using only 30% of the full acquisition data. These findings highlight sCNNs' potential for accurate and clinically efficient dMRI analysis, paving the way for improved diagnostic capabilities and characterization of early brain development with shorter scan times.

## 1 Introduction

Diffusion Magnetic Resonance Imaging (dMRI) is a non-invasive neuroimaging technique that provides unique insights into the microstructure of the brain and spinal cord tissue by measuring the diffusion of water molecules. By quantifying the directionality and magnitude of water diffusion, dMRI enables the mapping of white matter tracts and the characterization of microstructural changes associated with development (Snoussi et al., 2025; Karimi et al., 2024), aging (Luckey et al., 2024; Snoussi et al., 2023b), and various neurodegenerative diseases (Snoussi et al., 2023a). A typical dMRI acquisition involves acquiring a reference image with no diffusion weighting (*b* = 0 s/mm^2^) and a series of diffusion-weighted images. These are obtained by applying diffusion-sensitizing gradients in numerous orientations, represented by **q**-vectors that are carefully sampled across the surface of a sphere to capture the angular information of water diffusion within tissues.

Early identification of white matter abnormalities in neonates and accurately estimating microstructural parameters from dMRI are crucial for understanding brain architecture and identifying biomarkers for neurodevelopmental and neurological disorders (Kebiri et al., 2024). However, neonatal dMRI presents unique challenges, such as small brain size, low signal-to-noise ratio (SNR), motion artifacts, and ongoing myelination, that significantly hinder traditional analysis methods.

Traditional approaches to extracting microstructural information from dMRI, such as multi-shell multi-tissue constrained spherical deconvolution (MSMT-CSD) (Jeurissen et al., 2014), rely on fitting complex biophysical models to the dMRI signal. While effective with dense sampling, applying these methods to data acquired with a reduced number of diffusion directions often leads to less reliable Fiber Orientation Distribution (FOD) estimation due to increased noise sensitivity and model instability. This reliance on extensive data acquisition presents significant challenges for the healthcare system, increasing scanning costs, and limiting scanner throughput, thereby highlighting a critical clinical need for faster protocols.

Deep learning has emerged as a promising alternative for dMRI analysis, offering faster and potentially more robust parameter estimation (Karimi et al., 2021a, 2024; Kerkelä et al., 2024; Kebiri et al., 2024). Among these various methods, spherical convolutional neural networks (sCNNs) (Cohen et al., 2018; Esteves et al., 2018) have shown particular promise due to their inherent rotational equivariance. sCNNs are designed to be SO(3)-equivariant (i.e., rotating the input changes the output according to the same rotation) artificial neural networks that perform spherical convolutions with learnable filters. They enable rotationally equivariant processing of spherical data, making them well-suited for predicting microstructural parameters like the FOD from dMRI data.

While recent deep learning approaches, particularly sCNNs, have shown promise for dMRI analysis, their direct applicability to the unique challenges of neonatal imaging with highly constrained acquisition protocols remains largely unexplored. For instance, Kerkelä et al. (2024) explored sCNNs for general brain microstructure estimation using simulated and adult human data. Similarly, Sedlar et al. (2021) applied sCNNs to estimate Neurite Orientation Dispersion and Density Imaging (NODDI) parameters from adult Human Connectome Project (HCP) data, emphasizing the rotational equivariance of these networks. While these foundational studies highlight the power of sCNNs in handling the spherical nature of dMRI data and processing subsampled inputs, they generally focus on adult populations or scalar parameter estimation, and do not explicitly target the specific challenges of neonatal dMRI or the clinical implications of significantly reduced acquisition protocols for time-sensitive clinical use.

Other related works include those by Elaldi et al. (2024), who introduced an unsupervised rotation-equivariant spherical deconvolution framework for sparse FOD estimation. These methods leverage advanced spatial and spherical equivariance to improve deconvolution, but operate on an unsupervised training paradigm and are not primarily evaluated on neonatal data or the impact of significantly reduced acquisition protocols. Furthermore, while Karimi et al. (2021b) demonstrated that MLPs could estimate FODs from undersampled data with improved accuracy over traditional methods, their approach lacks the inherent rotational equivariance of sCNNs, which is critical for robust performance in diverse orientations.

**Figure 1:**
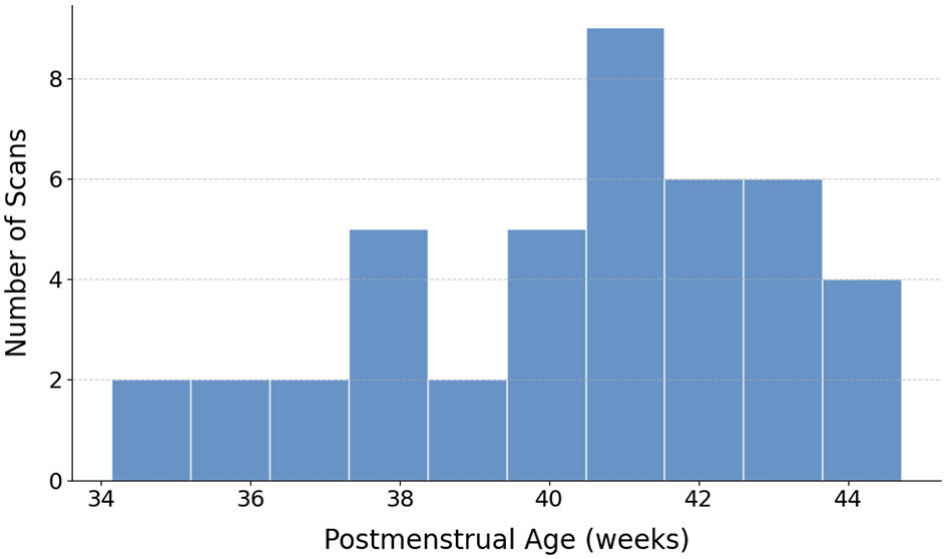
Distribution of the postmenstrual ages for the 43 neonatal dMRI datasets included in the study.

**Figure 2:**
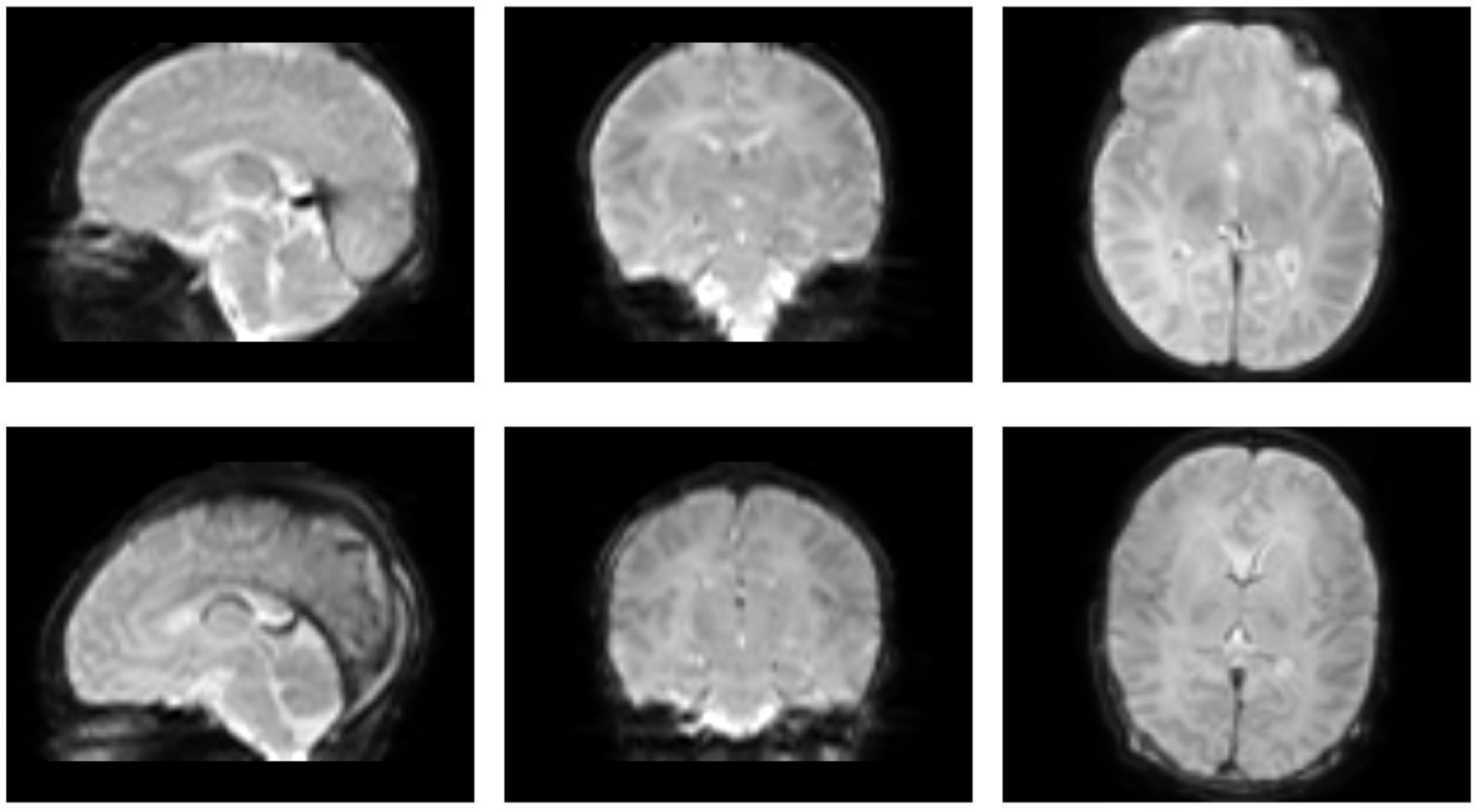
Sagittal, coronal, and axial views of representative examples of data from neonatal dMRI in the dHCP dataset.

In this work, we aim to bridge these gaps by developing and rigorously evaluating a novel sCNN framework uniquely tailored for the challenging domain of neonatal dMRI. Leveraging data from the Developing Human Connectome Project (dHCP) (see Figures 1, 2), our approach seeks to achieve accurate FOD estimation using a substantially reduced set of gradient directions. This directly addresses the critical need for faster, more feasible dMRI scans in neonates, which can significantly reduce scanning costs, improve patient comfort, and facilitate earlier diagnosis and intervention for neurodevelopmental disorders. We evaluate the performance of our framework using quantitative and qualitative metrics to demonstrate the downstream impact of accurate microstructural parameter estimation on connectomics analyses. The complete implementation, including training scripts, model architectures, and evaluation tools, is publicly available at: https://github.com/H-Snoussi/sCNN-FOD-neonatal.

## 2 Materials and methods

**Figure 3:**
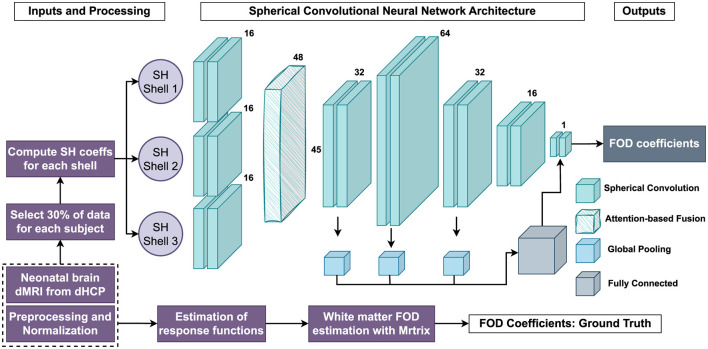
Flowchart illustrates the entire data processing and analysis pipeline, including the use of neonatal dMRI datasets, FOD estimation, data simulations, the sCNN architecture, and the outputs of the sCNN.

The methodology employed in this study encompasses several key stages, from data representation and preprocessing to model development, training, and evaluation. A comprehensive overview of the entire process, including the processing of neonatal dMRI datasets, FOD estimation, sCNN architecture, and the network's outputs, is presented in Figure 3.

### 2.1 Neonatal dMRI data acquisition and preprocessing

This study utilized a carefully selected subset of 43 high-quality neonatal dMRI datasets from the Developing Human Connectome Project (dHCP). This subset was chosen to ensure a representative distribution across postmenstrual ages (Figure 1) and to maintain manageable computational demands for our deep learning pipeline, collectively yielding over 4.5 million FOD samples for training, validation, and testing. Figure 2 provides two representative examples of the neonatal dMRI data.

The dHCP neonatal dMRI acquisition protocol was designed to optimize data acquisition for the unique properties of the developing brain. It employed a uniformly distributed set of gradient directions across three *b*-value shells (Edwards et al., 2022). The protocol comprised 20 volumes at *b* = 0 s/mm^2^, 64 volumes at *b* = 400 s/mm^2^, 88 volumes at *b* = 1000 s/mm^2^, and 128 volumes at *b* = 2600 s/mm^2^. The temporal ordering of the acquired directions was strategically planned to maximize efficiency, mitigate the risks of infant motion artifacts, and adhere to gradient duty cycle constraints. Data were acquired with in-plane resolution of 1.5 × 1.5 mm, and 3 mm slices with 1.5 mm overlap. Image reconstruction was performed using a dedicated algorithm (Hutter et al., 2018; Cordero-Grande et al., 2018).

The dataset underwent a comprehensive preprocessing pipeline, including denoising, brain masking, dynamic distortion correction, and slice-to-volume motion correction using a multi-shell spherical harmonics and radial decomposition (SHARD) representation (Christiaens et al., 2021). Simple intensity normalization was performed by setting negative values to zero and clipping high values at the 95th percentile.

### 2.2 Ground truth FOD estimation

Ground truth FODs were estimated using a hybrid multi-tissue constrained spherical deconvolution (Hybrid-CSD) approach implemented in MRtrix3 (Tournier et al., 2019). This approach decomposes the diffusion-weighted signal into contributions from white matter (WM), gray matter (GM), and cerebrospinal fluid (CSF) compartments (Jeurissen et al., 2014). Response functions for GM and CSF tissues were estimated using the *dhollander* algorithm (Dhollander et al., 2019).

Our choice of this hybrid strategy stems from observations tailored to neonatal dMRI. While GM and CSF response functions were estimated using the robust *dhollander* algorithm (Dhollander et al., 2019), the WM response function required a more specific approach. In our experience, Dhollander's default WM voxel selection often underperforms in neonatal data: regions expected to exhibit complex fiber crossings are underrepresented, as the anisotropic signal can be erroneously absorbed into the GM-like compartment. In contrast, for WM, we followed the iterative procedure for single-fiber voxel selection described by Tournier et al. (2013). This method reliably identifies high-FA, single-fiber WM voxels, particularly in coherent tracts such as the corpus callosum. We applied a fractional anisotropy (FA) threshold of 0.5 for initial WM versus GM-CSF separation; this higher threshold was selected after empirical testing (including values like 0.3 and 0.35), as it consistently produced more anatomically plausible tractography in our neonatal data.

From a modeling standpoint, multi-tissue CSD decomposes the diffusion signal as a linear combination of response functions from distinct tissue compartments (WM, GM, CSF), each convolved with their respective FODs. These compartments are assumed to be independent and separable in both signal characteristics and anatomical location. Therefore, the response functions do not necessarily need to be estimated jointly or via the same method, as long as each function accurately reflects the diffusion profile of its target tissue. The Dhollander method, on the other hand, is well-suited for unsupervised estimation of GM and CSF response functions using multi-shell data due to its design, which intentionally decouples WM, GM, and CSF voxel selection, making it amenable to modular integration. This combined approach ensures robust and anatomically faithful ground truth FODs for our neonatal dataset.

WM FODs obtained via this Hybrid-CSD approach were represented in the spherical harmonics (SH) basis up to (*lmax* = 8), yielding 45 SH coefficients per voxel. The neonatal WM FOD datasets were divided into training (35 subjects), validation (4 subjects), and testing (4 subjects) sets.

### 2.3 Generation of reduced dMRI training data

To facilitate faster and more cost-effective neonatal dMRI analysis, we generated training data using only the first 30% of the full dHCP acquisition protocol's gradient directions. This reduced protocol consisted of 19 volumes at *b* = 400 s/mm^2^ (compared to 64 in the full protocol), 26 volumes at*b* = 1000 s/mm^2^ (compared to 88), and 38 volumes at *b* = 2600 s/mm^2^ (compared to 128). The *b* = 0 s/mm^2^ volumes are not considered in the computation of SH. For each *b*-value shell, SH coefficients were extracted from the diffusion-weighted data up to *lmax* = 8, resulting in 45 SH coefficients per shell. A summary of the dataset splits and the number of diffusion directions for both ground truth and training data is provided in Table 1. As detailed in the table, our training data utilized 83 diffusion directions, a significant reduction from the 280 directions used for ground truth estimation.

**Table 1:** Summary of dataset splits and number of diffusion directions.

**Split**	**Subjects**	**Voxels**	**Diffusion MRI directions**	
			**Ground truth**	**Training**
Training	35	3,703,986	280	83
Validation	4	358,015	280	83
Testing	4	483,950	280	83

Ground truth FODs were estimated using the full acquisition (280 directions), while the sCNN was trained on reduced data (30%, 83 directions).

### 2.4 sCNN model for FOD estimation

The core of this study is a Spherical Convolutional Neural Network (sCNN) designed to estimate WM FOD from a reduced set of dMRI measurements. The sCNN architecture is optimized for spherical signals, leveraging spherical convolutions to exploit the rotational properties of diffusion signals. This approach ensures a more structured and efficient learning process, maintaining consistency across different orientations.

#### 2.4.1 sCNN architecture and shell attention mechanism

The proposed sCNN model is built upon a hierarchical, shell-specific feature extraction strategy, incorporating attention mechanisms to enhance feature fusion across different diffusion shells. The architecture is illustrated in Figure 3.

Shell-specific convolutions are applied independently to the input diffusion-weighted data at different shells using three spherical convolutional layers. Each layer extracts relevant features from its corresponding shell before passing them to the next stage. To improve feature integration across shells, a shell attention module is employed, assigning dynamic weights to different shells to enhance the learning of critical structures by prioritizing the most informative features.

Shell-specific convolutions are applied independently to the input diffusion-weighted data at different shells using three spherical convolutional layers. Each layer extracts relevant features from its corresponding shell before passing them to the next stage. To improve feature integration across shells, a shell attention module is employed, assigning dynamic weights to different shells to enhance the learning of critical structures by prioritizing the most informative features. Specifically, for each shell-specific feature map ${\text{X}}_{i}\in {\mathbb{R}}^{B\times 16\times C}$ (where *B* is the batch size and *C* is the number of spherical harmonic coefficients), global average pooling is applied across the SH dimension to form a 48-dimensional feature vector **z** ∈ ℝ^*B* × 48^ by concatenating **z** = [mean(**X**_1_), mean(**X**_2_), mean(**X**_3_)]. The resulting feature vector **z** ∈ ℝ^48^ is passed through a two-layer feedforward network to generate shell attention logits **l** ∈ ℝ^3^:


(1) $$\begin{array}{l} \text{l}={\text{W}}_{2}\cdot \sigma \left({\text{W}}_{1}\text{z}+{\text{b}}_{1}\right)+{\text{b}}_{2}, \end{array}$$

where ${\text{W}}_{1}\in {\mathbb{R}}^{24\times 48}$, ${\text{W}}_{2}\in {\mathbb{R}}^{3\times 24}$, and σ(·) is a Leaky Rectified Linear Unit (Leaky ReLU) non-linearity with negative slope 0.1. The attention weights **a** ∈ ℝ^3^ are then computed using the softmax function:


(2) $$\begin{array}{l} a_{i}=\frac{\exp\left(l_{i}\right)}{\sum_{j=1}^{3}\exp\left(l_{j}\right)}\;\text{for }i=1,2,3, \end{array}$$

ensuring that $\underset{i}{\sum }a_{i}=1$ and *a*_*i*_ ≥ 0. These weights are broadcast and applied multiplicatively to each shell-specific feature map before concatenation. This mechanism enables the model to assign higher importance to more informative shells on a per-sample basis, rather than treating all shells equally.

Following attention-guided fusion, the network applies a series of spherical convolutional layers in an encoder-decoder configuration with increasing feature channels: 16, 32, and 64. Leaky ReLU activation functions are applied after each layer to introduce non-linearity. The decoder progressively refines the feature representations using a symmetric series of spherical convolutions, which enhances feature retention and improves reconstruction quality. Finally, the processed feature maps are passed through fully connected layers with batch normalization and ReLU activations to enhance learning efficiency. The output layer produces 45 SH coefficients representing the estimated WM FODs.

#### 2.4.2 Rotationally equivariant spherical convolution layers

The foundational operation in our sCNN architecture is the spherical convolution, which is specifically designed to process functions defined on the sphere—such as the dMRI signal—while preserving rotational structure. In diffusion imaging, signals are naturally represented using SH, a basis for functions on the unit sphere. SH coefficients capture both the magnitude and directionality of signal variation, making them particularly well-suited for modeling fiber orientation distributions.

Mathematically, a spherical convolution between a function *f* and a filter *h* is defined as:


(3) $$\begin{array}{l} \left(f*h\right)\left(\text{x}\right)=\int_{\text{SO}\left(3\right)}\text{d}\text{R}f\left(\text{R}{\hat{\text{e}}}_{3}\right)h\left({\text{R}}^{-1}\text{x}\right), \end{array}$$

where **x** is a point on the sphere, ${\hat{\text{e}}}_{3}$ is the north pole unit vector, and **R** ∈ SO(3) denotes a rotation. This operation is equivariant to 3D rotations, meaning:


$$\text{If}f^{\prime }\left(\text{x}\right)=f\left({\text{R}}^{-1}\text{x}\right),\text{then}\left(f^{\prime }*h\right)\left(\text{x}\right)=\left(f*h\right)\left({\text{R}}^{-1}\text{x}\right),$$

so rotating the input results in a rotated output. This is a critical property for diffusion MRI analysis, where fiber orientations can vary arbitrarily in space.

In our implementation, the spherical convolution is performed directly in the SH domain. Each degree *l* is associated with a learnable scalar weight that is shared across all *m*-orders within that degree. This ensures that the operation is SO(3)-equivariant, as rotations in SH space only mix coefficients within the same degree. These weights are stored in a tensor of shape [*C*_out_, *C*_in_, *L*], where *L* is the number of SH degrees (restricted to even *l* for antipodal symmetry, as is standard in diffusion MRI). A degree expansion mask is used to broadcast these scalar weights to all orders *m*, and the convolution is applied using an efficient Einstein summation.

To introduce non-linearity while preserving spherical structure, SH coefficients are transformed to the spatial domain using the Inverse Spherical Fourier Transform (ISFT), followed by a Leaky ReLU activation and then mapped back to the SH domain using the forward the Spherical Fourier Transform (SFT). While this spatial-domain nonlinearity breaks strict SO(3) equivariance, it preserves approximate rotation-awareness and maintains compatibility with the SH-based structure of the data.

When the number of input and output channels match, a residual connection is applied, which is inherently equivariant since addition is commutative with rotation. Only even SH degrees are used (e.g., *l* = 0, 2, 4, …), reflecting the antipodal symmetry of diffusion signals and reducing unnecessary parameters.

In summary, our spherical convolution layers apply band-limited, degree-wise learnable weights in the SH domain, preserving SO(3)-equivariance. Approximate equivariant nonlinearities are applied via ISFT/SFT transformations, ensuring the network remains lightweight and robust to arbitrary signal orientations. This design enables biologically and physically informed feature learning, critical for accurate and generalizable fiber orientation estimation in dMRI.

#### 2.4.3 Spatial domain loss function for FOD reconstruction

Standard Mean Squared Error (MSE) loss, when applied directly to SH coefficients, is suboptimal for FOD reconstruction. This is because SH coefficients do not contribute equally to the reconstructed FOD. Lower-order coefficients primarily govern the overall magnitude or isotropic component, while higher-order coefficients capture finer angular details. Using a basic MSE loss treats all coefficients equally, potentially penalizing errors in higher-order coefficients less than errors in lower-order ones, even though the latter can have a more significant impact on the overall FOD shape. Therefore, a more nuanced approach is required. We propose a modified MSE loss calculated in the spatial domain, rather than the SH domain, to address this issue.

Specifically, given predicted SH coefficients **p** ∈ ℝ^*B* × 45^ and target SH coefficients **t** ∈ ℝ^*B* × 45^ for a batch of size *B* the loss function first reconstructs the FOD signals in the spatial domain using the ISFT:


(4) $$\begin{array}{l} {\text{p}}_{\text{FOD}}=\text{U}\text{p},\;{\text{t}}_{\text{FOD}}=\text{U}\text{t} \end{array}$$

where **U** ∈ ℝ^*N* × 45^ is the ISFT matrix mapping SH coefficients back to the spatial domain, *N* is the number of spatial points used to represent the FOD. The loss is then computed as the mean squared difference between the predicted and target FOD signals:


(5) $$\begin{array}{l} L_{\text{MSE}}=\frac{1}{NB}\overset{B}{\underset{i=1}{\sum }}\overset{N}{\underset{j=1}{\sum }}{\left({\text{p}}_{\text{FOD},ij}-{\text{t}}_{\text{FOD},ij}\right)}^{2} \end{array}$$

By computing the loss in the spatial domain rather than directly in the SH coefficient space, this approach ensures that model predictions are optimized for their impact on the reconstructed diffusion signal rather than just the coefficient magnitudes. This strategy improves the model's ability to generate accurate fiber orientation estimates.

#### 2.4.4 Training procedure

The training procedure of the sCNN model was designed to optimize convergence while preventing overfitting. The model was trained using the AdamW optimizer with an initial learning rate of 10*e*−4 and a weight decay of 10*e*−4. The learning rate was adjusted using a step-based scheduler with a decay factor of 0.5 every 17 epochs. To ensure stable training, gradient clipping was applied with a maximum norm of 10.0.

Training data consisted of diffusion-weighted images sampled from a reduced set of gradient directions, from which SH coefficients were extracted up to *lmax* = 8, resulting in 45 SH coefficients per voxel. The model was trained for 80 epochs for one hour. The MSE loss function was used, computed after transforming the SH coefficients into the spatial domain using ISFT.

### 2.5 Comparison with multi-layer perceptron

We compared the performance of the sCNN with a common deep learning network, Multi-Layer Perceptron (MLP) (Goodfellow et al., 2016). We trained an MLP with four fully connected layers (256 nodes each) followed by batch normalization and ReLU activations. The MLP took the normalized dMRI signals as input and output the spherical harmonic coefficients of the FOD. The MLP was trained using MSE as loss function and optimizer as the sCNN but required five times more training batches to ensure convergence due to its higher parameter count. Despite its simplicity, the MLP provided a baseline for assessing the effectiveness of spherical convolutions in capturing rotationally invariant features.

### 2.6 Evaluation metrics

To comprehensively evaluate the performance of the proposed sCNN model comparing to the ground truth and the baseline method, we employed a set of quantitative and qualitative metrics. These metrics were designed to assess both the accuracy of the estimated FODs and their downstream impact on WM tractography. The quantitative metrics include MSE, Angular Correlation Coefficient (ACC), and Structural Similarity Index Measure (SSIM), which evaluate how closely the predicted FODs match the ground truth in both coefficient and angular space. Additionally, we conducted tractography-based assessments to evaluate the practical implications of FOD quality on the reconstruction of WM pathways.

#### 2.6.1 Mean squared error

The MSE was used as the primary metric to quantify the discrepancy between the predicted and reference FODs. For each voxel, the MSE was computed directly in the SH domain as the mean squared difference between the predicted and ground truth SH coefficients:


$$\text{MSE}=\frac{1}{N}\overset{N}{\underset{i=1}{\sum }}||{Ŝ}_{i}-S_{i}{||}^{2},$$

where Ŝ_*i*_ and *S*_*i*_ are the predicted and ground truth SH coefficient vectors for voxel *i*, and *N* is the number of voxels.

#### 2.6.2 Angular correlation coefficient

The Angular Correlation Coefficient (ACC) measures the similarity in orientation between predicted and ground truth FODs in the spatial domain (Anderson, 2005). For each voxel, FODs are reconstructed by projecting SH coefficients onto a dense spherical grid. ACC is then calculated as the cosine similarity between the reconstructed FODs:


$$\text{ACC}=\frac{\langle F\hat{O}D,FOD\rangle }{||F\hat{O}D||\cdot ||FOD||},$$

where $F\hat{O}D$ and *FOD* represent the predicted and ground truth FOD amplitudes over the sphere. A higher ACC value indicates better alignment of fiber orientations.

#### 2.6.3 Structural similarity index measure

The Structural Similarity Index (SSIM) is a perceptual metric that quantifies image similarity by evaluating three key aspects: luminance patterns, contrast relationships, and structural composition. For FOD evaluation, we compute SSIM independently for each spherical harmonic coefficient channel. This channel-wise approach preserves harmonic-specific spatial information and provides a comprehensive assessment of reconstruction fidelity across all angular frequencies present in the FOD field.

#### 2.6.4 Angular error

Angular Error (AE) quantifies the average angular deviation between the primary peak directions of the predicted and ground truth FODs. For each voxel, the directions of the principal fiber orientation are identified from the reconstructed FODs. The angular error is then computed as the angle between these corresponding peak directions, averaged across all valid voxels. A lower AE indicates higher accuracy in fiber orientation estimation.

#### 2.6.5 Peak match rate

Peak Match Rate (PMR) represents the percentage of voxels where the angular error between the principal peaks of the predicted and ground truth FODs falls below a predefined threshold (e.g., 20°). This metric indicates the proportion of voxels where the primary fiber direction is accurately reconstructed within an acceptable angular tolerance. A higher PMR signifies better fidelity in resolving fiber orientations.

#### 2.6.6 Peak signal-to-noise ratio

PSNR is a widely used metric to quantify the quality of reconstruction, representing the ratio between the maximum possible power of a signal and the power of corrupting noise. A higher PSNR value indicates a better quality reconstruction.

#### 2.6.7 Tractography-based evaluation

To assess the downstream utility of the predicted FODs, we performed probabilistic tractography using the iFOD2 algorithm (Tournier et al., 2010) implemented in MRtrix3 (Tournier et al., 2019). Streamlines were generated with dynamic seeding based on FOD amplitude, uniformly distributed throughout a white matter mask. Anatomical constraints were imposed using a 5-tissue-type (5TT) image generated from structural data. This approach guides streamline propagation and improves anatomical realism. Specific tractography parameters included a select limit of 100,000 streamlines, an FOD amplitude cutoff of 0.001, a step size of 0.4 mm, a maximum angle of 20 degrees, and streamline length constraints between 5 mm and 300 mm. The resulting tractograms were then visually inspected for anatomical plausibility, coherence, and coverage of major white matter bundles. This qualitative evaluation helps determine whether differences in FOD estimation affect tract reconstruction.

### 2.7 Implementation details and code availability

The sCNN and MLP models were implemented using PyTorch (Paszke et al., 2019) and trained on an NVIDIA RTX A6000 GPU with 48 GB of memory. Training the sCNN model required approximately 1.1 h, while the MLP model required approximately 6 h. The source code, trained models, and scripts for reproducing the results are publicly available at https://github.com/H-Snoussi/sCNN-FOD-neonatal.

## 3 Experiments and results

### 3.1 Quantitative evaluation of FOD estimation accuracy

**Figure 4:**
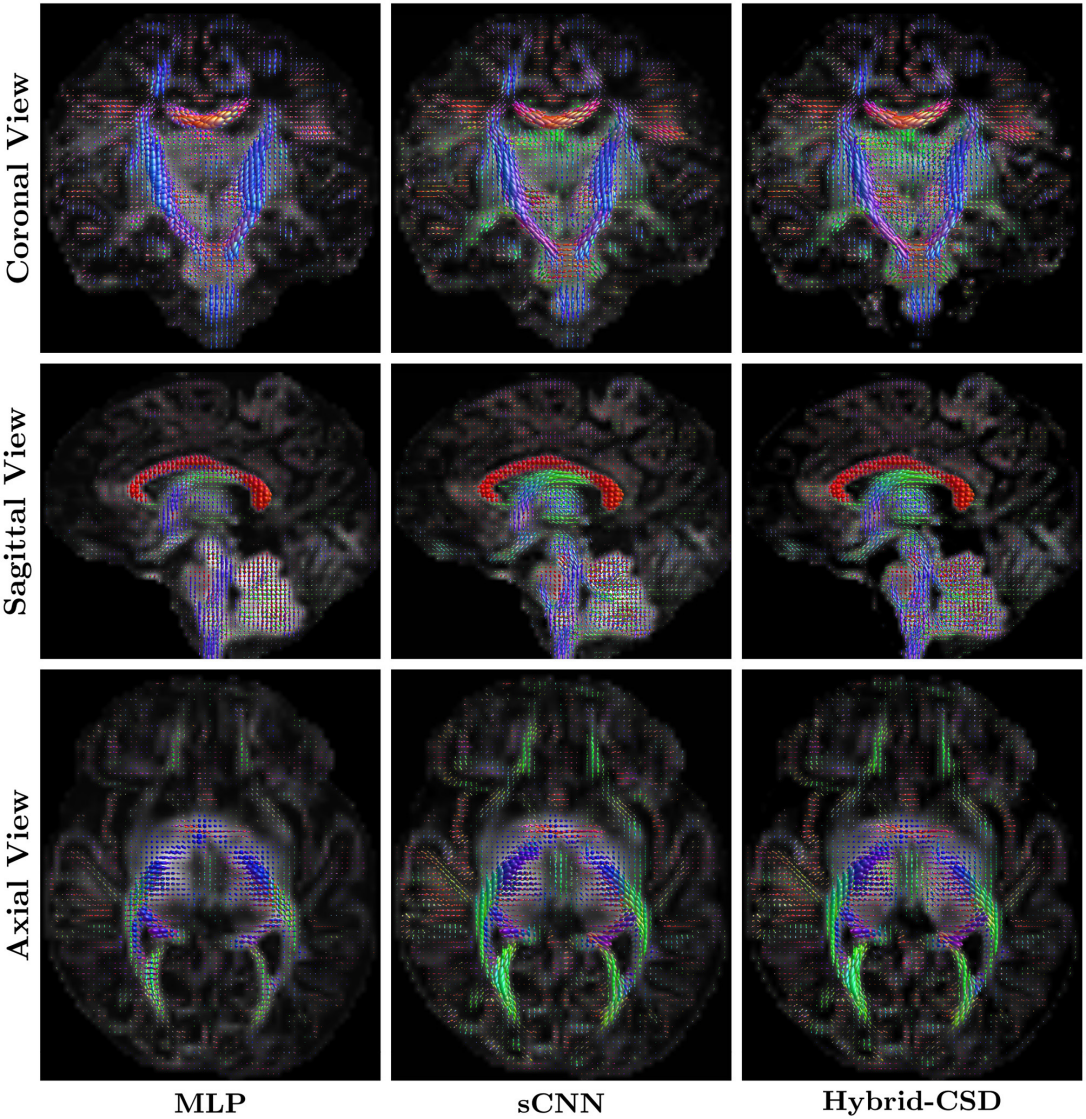
Representative FODs from a test subject. **(Left column)** FODs estimated by the MLP using the full dHCP dataset. **(Middle column)** FODs estimated by the sCNN using 30% of the diffusion directions. **(Right column)** Ground truth FODs estimated using Hybrid-CSD with the full dHCP dataset. The sCNN produces FODs that are visually much more similar to the ground truth than the MLP.

**Figure 5:**
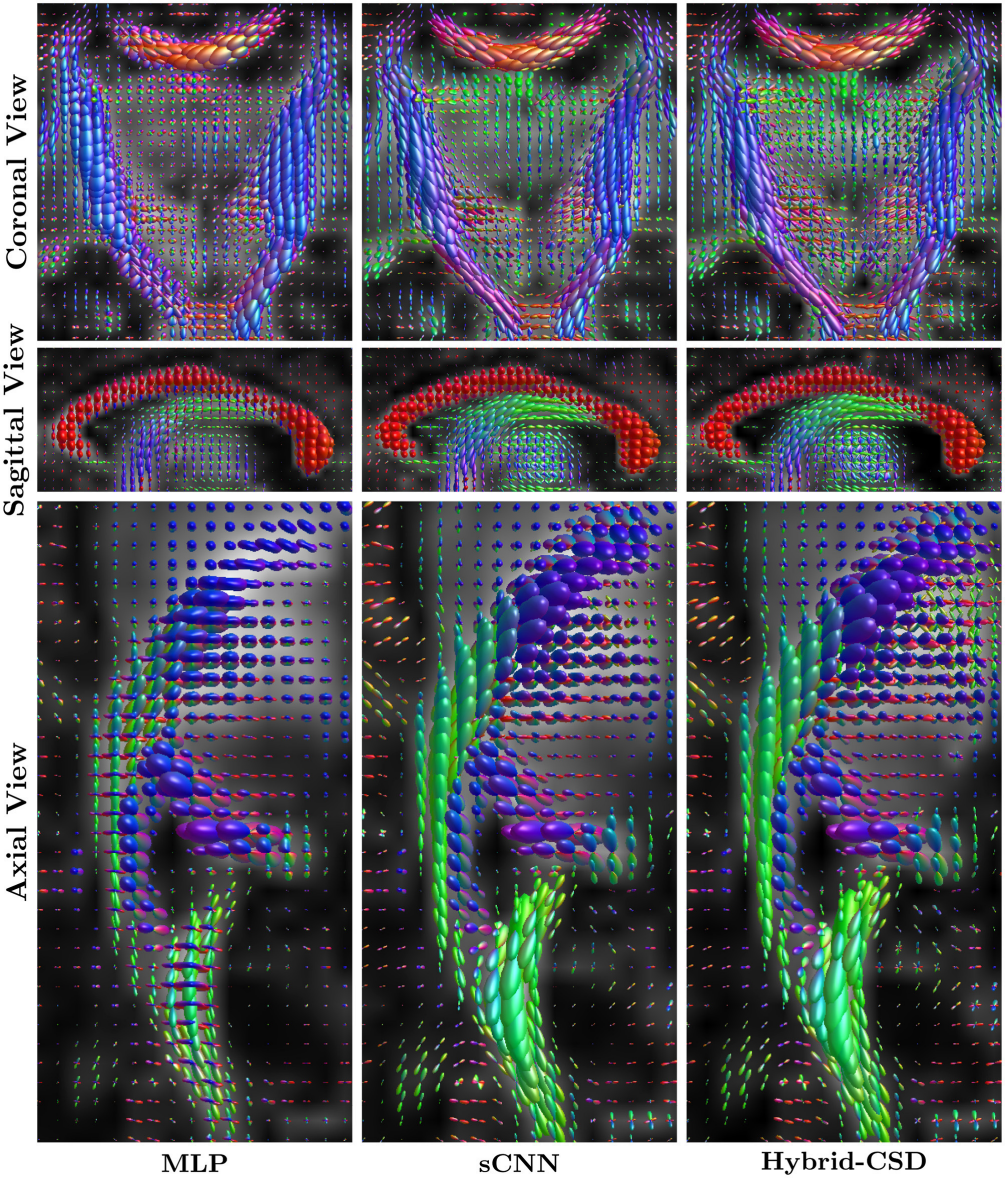
Zoomed-in views of regions of interest (ROIs) with complex fiber configurations, highlighting differences between FODs predicted by MLP, sCNN, and Hybrid-CSD (ground truth). The sCNN preserves anatomical structure and closely resembles the ground truth, whereas the MLP exhibits increased noise and reduced structural clarity. These ROIs correspond to those shown in Figure 4.

**Figure 6:**
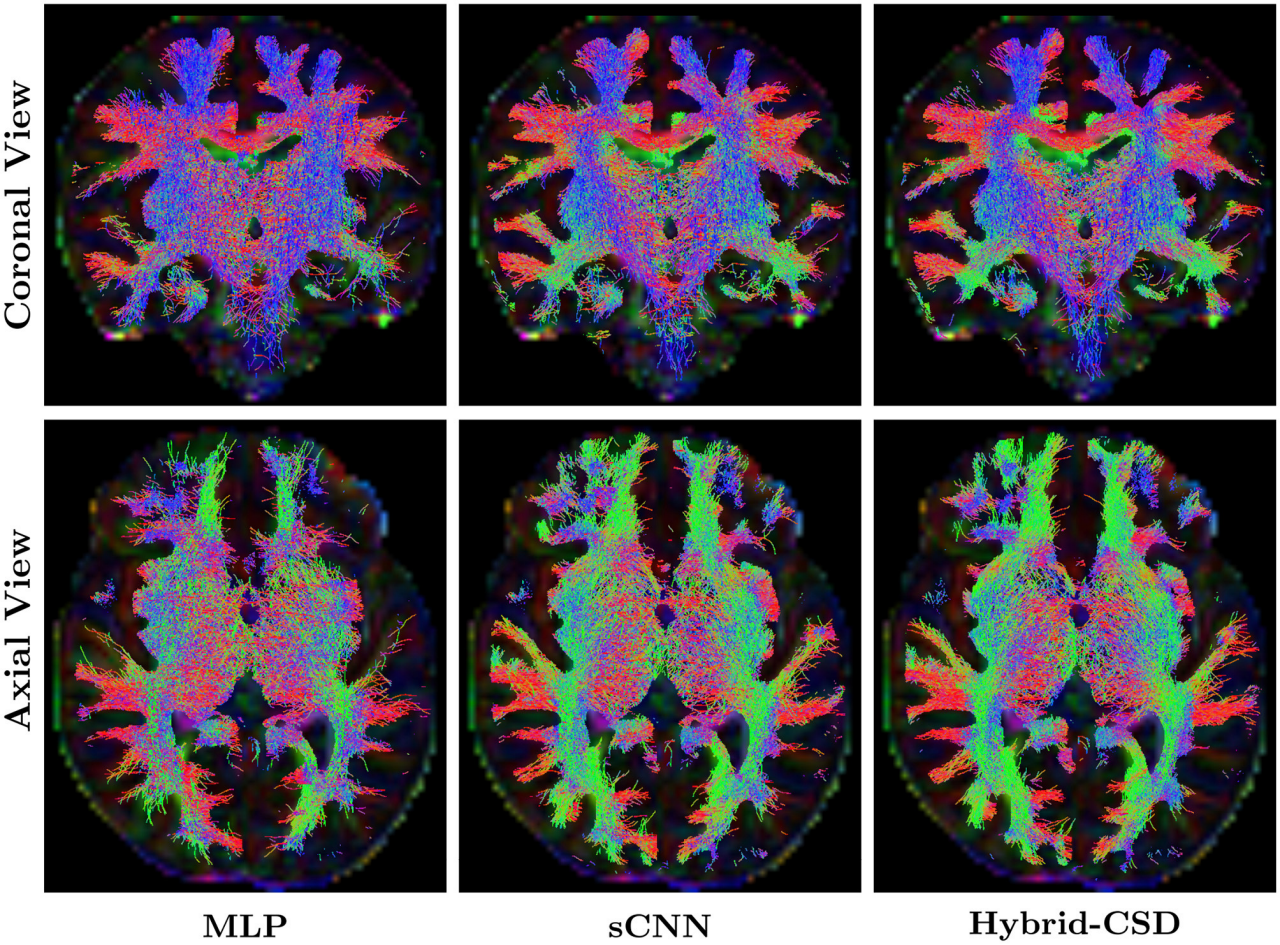
Representative tractography results. **(Left)** Tractogram generated using MLP-predicted FODs. **(Middle)** Tractogram generated using sCNN-predicted FODs. **(Right)** Tractogram generated using ground truth FODs (Hybrid-CSD).

Table 2 presents the quantitative results for FOD estimation, comparing the sCNN and MLP models against the ground truth (Hybrid-CSD) on a representative subject from the test set (the same subject used in Figures 4–6). The rotationally equivariant sCNN significantly outperformed the MLP in all metrics, with all paired comparisons yielding *p* < 0.001 for the FOD-wise metrics (statistical details provided in the table caption). For the global metrics which are computed over the entire masked region, sCNN reduced the Mean Squared Error (MSE) from 0.0012 to 0.0001 (-91.7%), raised the Peak Signal-to-Noise Ratio (PSNR) from 22.31 dB to 34.40 dB, and likewise increased the SSIM from 0.904 to 0.977. Regarding the FOD-wise metrics, the ACC rose from 0.773 ± 0.132 to 0.984 ± 0.084, and the Mean Angular Error fell from 66.86 ± 66.53° to 6.26 ± 24.30°. Peak-match rate within 20° climbed from 0.500 ± 0.007 to 0.970 ± 0.003. These gains confirm the sCNN's superior ability to recover complex fiber orientations.

**Table 2:** Quantitative evaluation of FOD estimation for a representative subject from the test set.

**Category**	**Metric**	**MLP**	**sCNN**
Global scalar	MSE	0.0012	0.0001
	SSIM	0.9043	0.9770
	PSNR (dB)	22.310	34.402
FOD-wise	ACC	0.773 ± 0.132	0.984 ± 0.084
	Peak match (20°)	0.500 ± 0.007	0.970 ± 0.003
	Mean ang. error (°)	66.86 ± 66.53	6.26 ± 24.30

FOD-wise metrics show statistically significant improvements for sCNN over MLP, *p* < 0.001. Statistical significance for continuous metrics (ACC, Mean Angular Error) was assessed using paired *t*-tests, and for Peak Match Rate using McNemar's test. Lower values are better for MSE and Mean Angular Error.

### 3.2 Qualitative FOD visualization

Figures 4, 5 present visual comparisons of the FODs estimated by the sCNN, MLP, and Hybrid-CSD (ground truth) for an example of a test subject. Visually, the sCNN-predicted FODs closely resemble those generated by Hybrid-CSD, demonstrating clear and anatomically consistent fiber peaks with reduced noise and spurious orientations. In contrast, the MLP-predicted FODs appear notably less accurate, often exhibiting a lack of clear directional coherence in major white matter regions such as the corpus callosum and the corticospinal tract. A common artifact observed in the MLP results is the presence of spurious crossing fibers in voxels that should predominantly exhibit a single, coherent direction.

The sCNN FODs consistently show sharper peaks and better delineate fiber orientations. Figure 5 provides a zoomed-in view of specific regions of interest (ROI) to further highlight the superior performance of the rotationally equivariant sCNNs in preserving structural fidelity and resolving complex fiber architectures.

### 3.3 Tractography analysis

Tractography, while a powerful tool for visualizing white matter pathways, is inherently sensitive to the quality of the underlying FOD estimates. Figure 6 shows representative tractography results generated from the FODs produced by each method for a test subject. The sCNN-based tractograms, derived from the reduced acquisition data, demonstrate a high degree of visual similarity and anatomical plausibility when compared to the Hybrid-CSD ground truth tractography. The sCNN successfully reconstructs major white matter pathways, such as the corpus callosum and the corticospinal tract, with greater fidelity and fewer spurious streamlines than the MLP. In contrast, the MLP tractogram exhibits considerable noise and largely fails to accurately represent these key pathways, showing a clear lack of directional coherence and anatomical fidelity.

## 4 Discussion

This study demonstrates the significant potential of rotationally equivariant sCNN for accurate and efficient FOD estimation in neonatal dMRI, using a substantially reduced acquisition protocol. Our sCNN approach produces results that are quantitatively comparable and qualitatively highly similar to those obtained using a reliable Hybrid-CSD ground truth, despite utilizing only 30% of the full acquisition data. This striking finding, which we elaborate on below, stems from the sCNN's ability to learn a robust representation that effectively mitigates the known limitations of model-based approaches in challenging neonatal data. These findings have important implications for the analysis of neonatal dMRI data and its potential for earlier, more accurate, and more efficient diagnosis of neurodevelopmental disorders.

### 4.1 FOD estimation accuracy and fidelity

Our quantitative results (Table 2) demonstrate that the proposed sCNN model achieves significantly superior Fiber Orientation Distribution (FOD) estimation accuracy compared to the MLP baseline across a comprehensive set of metrics, including MSE, SSIM, PSNR, ACC, Mean Angular Error (MAE), and Peak Match Rate (PMR). These substantial improvements highlight the effectiveness of the sCNN's architecture in reconstructing FODs from reduced diffusion data.

Beyond quantitative measures, qualitative visual inspection (Figures 4, 5) provides compelling evidence of the sCNN's enhanced FOD fidelity. The sCNN-predicted FODs consistently exhibit sharper peaks, better delineate fiber orientations, and show a clear reduction in noise and spurious orientations compared to the MLP. Notably, in regions of complex fiber configurations, the sCNN maintains structural fidelity and resolves these architectures more effectively, while the MLP often produces less accurate FODs with a lack of directional coherence and spurious crossing fibers in seemingly unidirectional voxels.

### 4.2 Model design and performance drivers

The performance of the sCNN is attributable to several key factors inherent to its design. First, the sCNN's core property of rotational equivariance ensures that it learns features that are intrinsically invariant to the orientation of the head within the scanner. This is a fundamental requirement for dMRI analysis, as the diffusion signal's orientation directly reflects the underlying fiber orientation. The MLP, lacking this built-in equivariance, must learn rotational invariance from the data, which is a more challenging task that typically requires larger datasets and more complex model architectures. Second, the shell attention mechanism allows the sCNN to dynamically weight the contributions of different *b*-value shells, which may vary depending on the degree of myelination. Third, the use of spherical convolutions allows the sCNN to operate directly on the SH representation of the diffusion signal. This avoids the need for interpolation or resampling, which can introduce artifacts and degrade the accuracy of FOD estimation. Fourth, the spatial-domain loss function, computed after transforming the SH coefficients to the spatial domain, emphasizes perceptually all SH orders without neglecting the finer angular details captured by lower-order coefficients. This ensures that the model optimizes for the shape of the FOD, not just the SH coefficient values. Our model applies spherical convolutions in the SH domain on a voxel-wise basis, without incorporating spatial information from neighboring voxels. Therefore, the model does not introduce spatial smoothing across voxels. Instead, it learns to denoise FODs by operating directly on the SH representation, capturing consistent angular patterns while suppressing noise.

### 4.3 Tractography and diagnostic quality

The tractography results, presented in Figure 6, highlight the downstream utility of the predicted FODs. sCNN-based tractography, generated from only 30% of the acquisition data, demonstrates a high degree of visual similarity and anatomical plausibility when compared to the Hybrid-CSD ground truth tractography. Specifically, we observe similar tract configurations and coherence in major white matter pathways such as the corticospinal tract, the corpus callosum (particularly visible in the coronal view), and the superior corona radiata (axial view). It is important to note that, as discussed by Pietsch et al. (2019), the Hybrid-CSD FODs, while a widely accepted model-based reference, possess inherent limitations in the challenging neonatal context. These include difficulties in accurately separating tissue types and fully resolving complex fiber configurations in immature brains, often leading to sparse or noisy representations.

In contrast, the MLP-based tractography appears relatively noisy, with some directions and colors of tracts inconsistent with those observed in the Hybrid-CSD reference. This indicates a clear lack of directional coherence and anatomical fidelity compared to both the sCNN and the ground truth. The sCNN's superior performance, therefore, suggests that it is capable of effectively denoising the diffusion signal and learning a more robust representation of the underlying white matter architecture, which translates into more reliable tractography.

### 4.4 Shell-attention mechanism and multi-shell robustness

Our sCNN architecture was specifically designed to optimally leverage multi-shell diffusion data, incorporating shell-attention layers that learn to adaptively weight the contribution of each shell during training. In our internal experiments, removing this shell-attention mechanism led to a clear degradation in performance, underscoring the importance of multi-shell input for robust FOD estimation. While our primary training and evaluation were conducted on reduced, multi-shell data (83 volumes), this shell-attention mechanism also offers a degree of flexibility: if only a single shell is available, the model can still be applied using the corresponding learned weights for that shell.

### 4.5 Clinical impact and translational relevance

The fact that accurate FOD estimation and tractography can be achieved using only 30% of the full dHCP acquisition protocol has substantial practical implications. Reducing scan time is crucial in neonatal imaging, as it improves patient comfort, minimizes the risk of motion artifacts, and increases scanner availability, making dMRI more accessible for routine clinical use. This finding underscores the sCNN's ability to extract more information from a limited amount of data, a critical advantage in challenging imaging scenarios. Our reduced acquisition protocol holds strong potential for enabling unsedated scanning during natural sleep cycles, critical for monitoring preterm infants at risk for cerebral palsy. This could triple scanner throughput in the Neonatal Intensive Care Units (NICUs) while reducing parental anxiety from prolonged separations.

Beyond the immediate application to neonatal dMRI, our findings suggest that sCNNs have broader potential for improving dMRI analysis in other populations and applications. The challenges of motion artifacts and scan time constraints are even more pronounced in fetal and pediatric dMRI, making the sCNN approach potentially even more valuable in these contexts. The public release of our training pipeline, including the trained models and data processing scripts, facilitates the rapid translation of this methodology to other vulnerable populations and encourages further research in this area.

### 4.6 Limitations and future work

Despite these promising results, this study has some limitations. While the sample size of 43 subjects is larger than many previous studies in dMRI, future work should validate these findings on larger, more diverse datasets, including subjects with different clinical conditions.

Second, while Hybrid-CSD remains a reliable approach for FOD estimation, its application to neonatal data presents known limitations (Pietsch et al., 2019). Specifically, Hybrid-CSD can struggle in regions of complex fiber architecture and in accurately separating tissue compartments, a concern well-documented in recent literature. Distinguishing tissue types like GM and WM based on signal decay is particularly challenging in neonates, where the average signal in cortical GM can be nearly indistinguishable from parts of the corpus callosum, and WM signal characteristics exhibit strong age dependence. These evolving microstructural properties suggest that models based on fixed response functions or those optimized for mature brains may not fully capture the complex, developing properties of neonatal tissue. Third, the reduced acquisition protocol used in this study (30% of directions) was chosen empirically. Future work should investigate the optimal acquisition protocol for sCNN-based FOD estimation, potentially using active learning strategies to identify the most informative diffusion directions. Fourth, while our goal was not to introduce a novel sCNN architecture, future studies could benefit from a more extensive comparison to alternative spherical CNN models and an ablation study of architectural components. Such analyses would provide a deeper understanding of which aspects of the network architecture most influence performance in neonatal FOD reconstruction.

## 5 Conclusion

This study contributes to the growing body of research on deep learning for medical image analysis by demonstrating the feasibility and potential of sCNNs for accurate and efficient FOD estimation in neonatal dMRI. The proposed sCNN model outperforms a standard MLP in terms of both quantitative metrics and tractography results, highlighting the benefits of rotational equivariance and shell-specific processing. The ability to achieve accurate FOD estimation with a reduced acquisition protocol has significant implications for clinical practice, potentially leading to faster, more cost-effective, and less burdensome neonatal dMRI scans. This research paves the way for improved characterization of early brain development and earlier, more accurate diagnosis of neurodevelopmental disorders, contributing to improved clinical outcomes for vulnerable neonatal populations.

## Data Availability

Publicly available datasets were analyzed in this study. This data can be found here: https://www.developingconnectome.org.
